# Supplementary material for: Interneuron-specific plasticity at parvalbumin and somatostatin inhibitory synapses onto CA1 pyramidal neurons shapes hippocampal output
Source: Nat Commun. 2020 Sep 2;11:4395. doi: 10.1038/s41467-020-18074-8 (PMC7467931; doi:10.1038/s41467-020-18074-8)
Supplement: Supplementary file 1 — Supplementary Information [file 41467_2020_18074_MOESM1_ESM.pdf]

Interneuron-specific plasticity at parvalbumin and somatostatin inhibitory synapses onto CA1 pyramidal neurons shapes hippocampal output

Udakis et al.

Supplementary Figures 1-7

Supplementary Figure 1

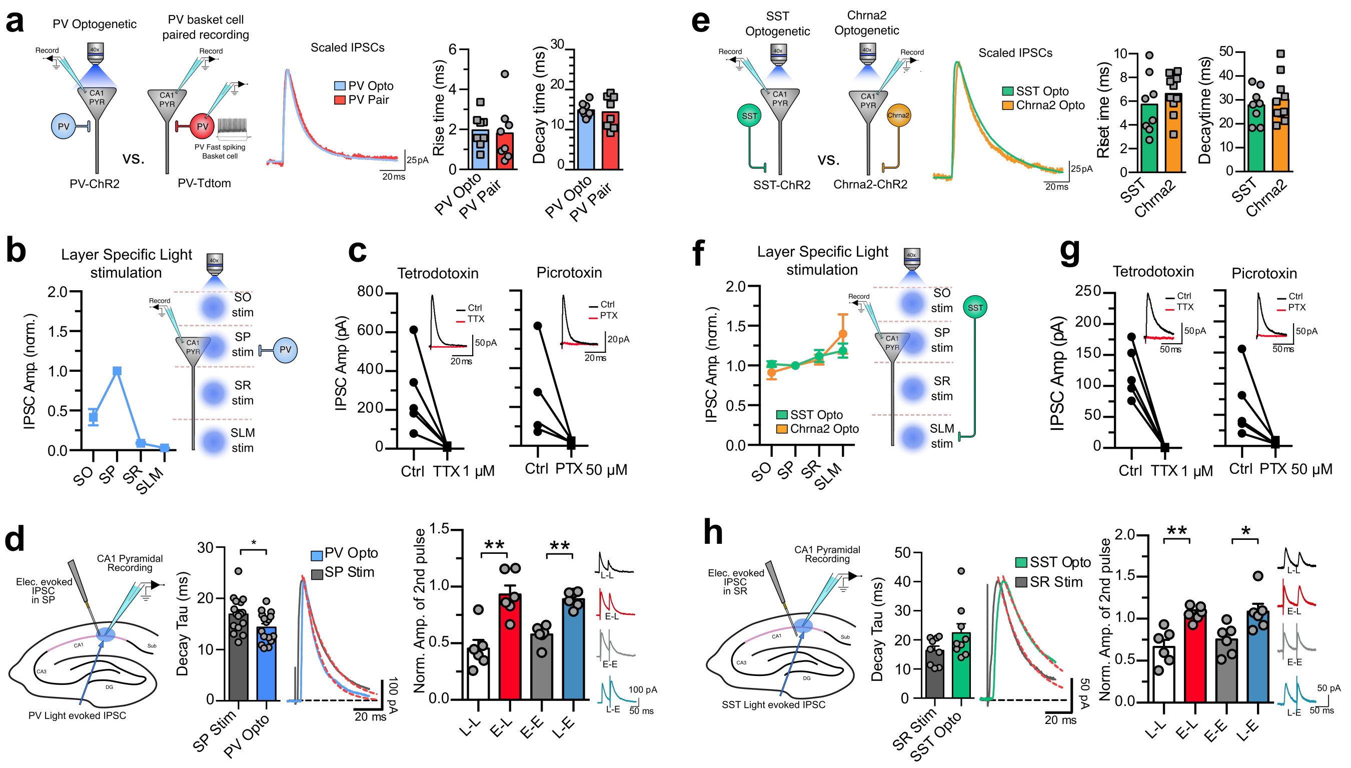

Supplementary Figure 1 (related to Fig. 1). Characterisation of PV and SST optogenetic responses.

(a) Comparison of optogenetically evoked PV IPSCs and IPSCs recorded via PV fast-spiking basket cell and CA1 pyramidal neuron paired recordings. Both rise and decay times were indistinguishable PV-ChR2; n = 8 cells PV-Pair; n = 8 cells) **(b)** PV IPSCs evoked via light stimulation through the objective lens positioned over different layers of the hippocampus. PV IPSC amplitude was highest in the pyramidal layer consistent with PV basket cell activation, n = 4 cells. **(c)** PV IPSCs were completely blocked by 1µM tetrodotoxin and 50µM picrotoxin showing optogenetic IPSCs are action potential and $GABA_A$ receptor dependent, TTX; n = 5 cells, PTX n = 4 cells. **(d)** Comparison of the decay kinetics between PV-optogenetic light-evoked pathway and electrically-evoked IPSC control pathway in SP, (P = 0.0278, unpaired t-test, two-tailed, n = 16 cells). (Right) Check of pathway independence, electrical stimulation failed to depress light responses whilst light responses failed to depress electrical responses indicating separate discrete inhibitory synapse activation. (L-L vs E-L; P = 0.0079, L-E vs E-E; P = 0.0013, paired t-tests, two-tailed, n = 6 cells) **(e)** Comparison of optogenetically evoked IPSCs SST and Chrna2 expressing OLM interneurons. Both rise and decay times were indistinguishable SST-ChR2; n = 8

cells Chrna2-ChR2; n = 10 cells). **(f)** SST and Chrna2 IPSCs evoked via light stimulation of different layers of the hippocampus. Both SST and Chrna2 IPSC amplitudes were maintained across all layers and highest in stratum lacunosum moleculare layer consistent with SST OLM interneuron activation. SST; n = 3 cells, Chrna2; n = 6 cells **(g)** SST IPSCs were completely blocked by 1μM tetrodotoxin and 50μM picrotoxin showing optogenetic IPSCs are action potential and $GABA_A$ receptor dependent, , TTX; n = 5 cells, PTX n = 5 cells. **(h)** Comparison of the decay kinetics between SST-optogenetic light-evoked pathway and electrically-evoked IPSC control pathway in the SR (P = 0.101, unpaired t-test, two-tailed, n = 9 cells). (Right) check of pathway independence, electrical stimulation failed to depress light responses whilst light responses failed to depress electrical responses indicating separate discrete inhibitory synapse activation. (L-L vs E-L; P = 0.0062, L-E vs E-E; P = 0.0124, paired t-tests, two-tailed, n = 6 cells) Data presented as mean values ± S.E.M.

Supplementary Figure 2

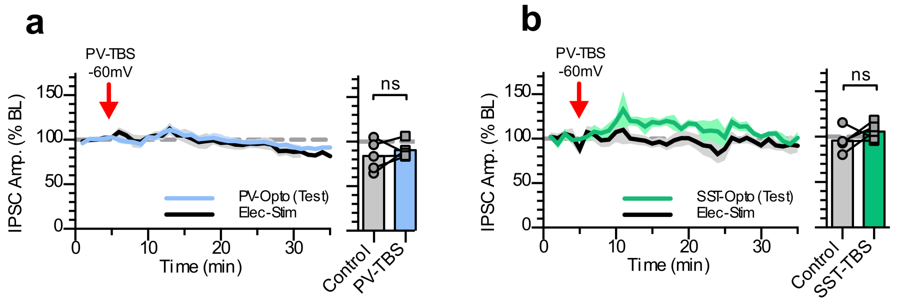

Supplementary Figure 2 (related to Fig. 1). TBS induced PV-iLTD and SST-iLTP are dependent on postsynaptic depolarisation

CA1 pyramidal neurons were recorded at 0 mV for the duration of the experiment except for during the light induced TBS protocol in which the neuron was held at -60 mV. **(a)** PV-iLTD was not induced if CA1 pyramidal neurons were held at -60 mV during the induction protocol. (P = 0.434, paired t-test, two-tailed, n = 5 cells) **(b)** SST-iLTP failed to be induced when CA1 pyramidal neurons were held at -60 mV during the induction protocol. (P = 0.290, paired t-test, two-tailed, n = 5 cells). Data presented as mean values ± S.E.M .

Supplementary Figure 3

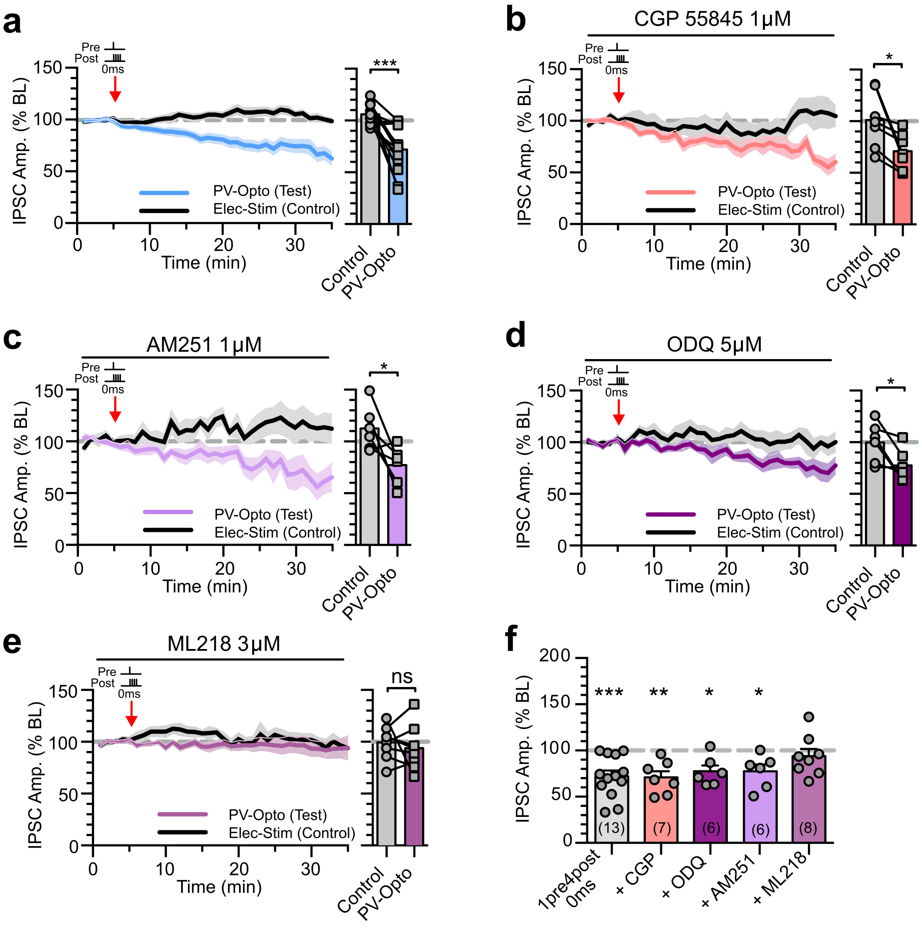

Supplementary Figure 3 (related to Fig. 3). PV-iLTD is not dependent on endocannabinoid, GABA$_B$ receptors or nitrous oxide signalling.

(a) PV-iLTD induced by 0 ms 1pre 4post timing (data from Fig. 2c). (P = 0.0003, paired t-test, two-tailed, n = 13 cells) **(b)** GABA$_B$ antagonist CGP55845 (1 μM) failed to block PV-iLTD. (P = 0.0244, paired t-test, two-tailed, n = 7 cells) **(c)** CB1 receptor antagonist AM251 (1 μM) failed to block PV-iLTD. (P = 0.0394, paired t-test, two-tailed, n = 6 cells) **(d)** Inhibiting the Nitrous oxide pathway via inhibition of guanylyl cyclase with ODQ (5 μM) failed to block PV-iLTD. (P = 0.0322, paired t-test, two-tailed, n = 6 cells) **(e)** The selective T-type VGCC inhibitor ML218 (3 μM) blocked PV-iLTD (P = 0.5301, paired t-test, two-tailed, n = 8 cells) **(f)** Summary histogram displaying the level of plasticity under each experimental condition, significance refers to paired t-tests in (a-e). Data presented as mean values ± S.E.M.

Supplementary Figure 4

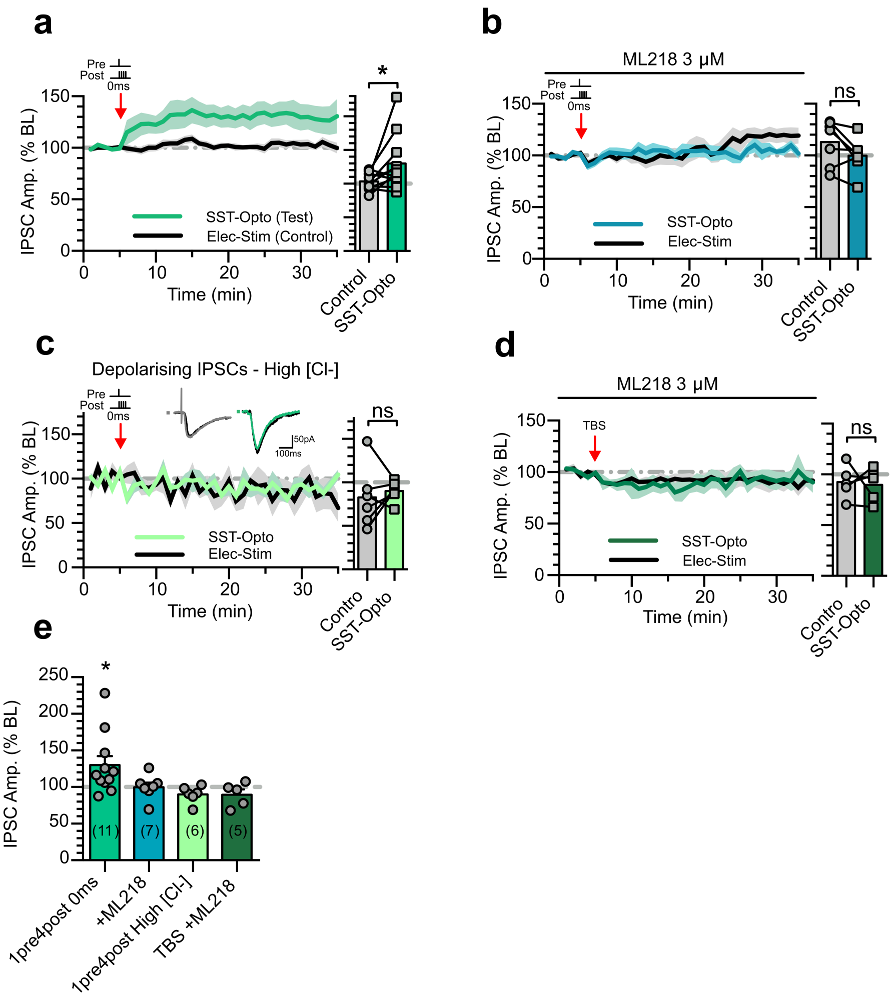

Supplementary Figure 4 (related to Fig. 4). SST-iLTP requires T-type VGCC activation.

(a) SST-iLTP induced by 0 ms 1pre 4post timing (data from Fig. 2g). (P = 0.0481, paired t-test, two-tailed, n = 11 cells) **(b)** The selective T-type VGCC inhibitor ML218 (3 μM) blocked SST-iLTP. (P = 0.0727, paired t-test, two-tailed, n = 7 cells) **(c)** Depolarising IPSCs caused by high [Cl$^-$] (50mM) internal solution blocked SST-iLTP. Inset example IPSCs showing inward current. (P = 0.613, paired t-test, two-tailed, n = 6 cells) **(d)** ML218 (3 μM) blocked SST-iLTP induced by theta burst synaptic stimulation during membrane depolarisation. (P = 0.789, paired t-test, two-tailed, n = 5 cells) **(e)** Summary histogram displaying the level of plasticity under each experimental condition, significance refers to paired t-tests in (a-d). Data presented as mean values ± S.E.M.

Supplementary Figure 5

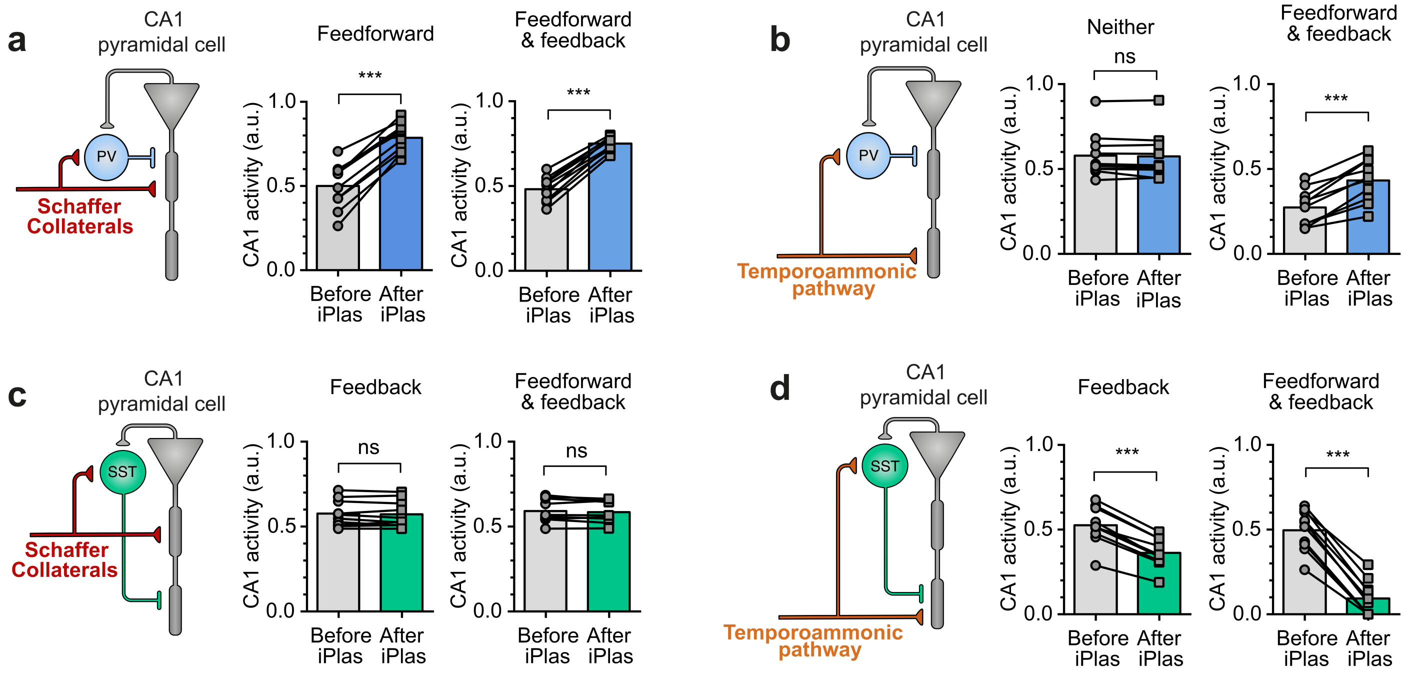

Supplementary Figure 5 (related to Fig. 6). PV and SST plasticity regulate CA1 pyramidal neuron excitability via feedback and feedforward inhibition.

(a) PV-iLTD increased CA1 pyramidal neuron activity in responses to Schaffer collateral input if PV interneurons are engaged via feedforward inhibition or feedforward and feedback inhibition. (Feedforward; P < 0.0001, Feedforward & feedback; P < 0.0001, paired t-tests, two-tailed, n = 10). **(b)** PV-iLTD had no effect on temporoammonic excitation of CA1 pyramidal neurons due to lack of feedforward or feedback inhibition. (P = 0.391, paired t-test, two-tailed, n = 10). If the temporoammonic pathway recruits PV interneurons via feedforward or partake in feedback inhibition PV-iLTD increased temporoammonic pathway driven CA1 activity. (P < 0.0001, paired t-test, two-tailed, n = 10). **(c)** SST-iLTP had no effect on the Schaffer collateral induced CA1 pyramidal neuron excitability if it is engaged via feedback or feedback and feedforward inhibition. (Feedback; P = 0.572, Feedforward & feedback; P = 0.154, paired t-tests, two-tailed, n = 10). **(d)** SST-iLTP reduced the temporoammonic pathway driven excitability of CA1 pyramidal neurons when engaged via feedback or feedforward and feedback inhibition. (Feedforward; P < 0.0001, Feedforward & feedback; P < 0.0001, paired t-tests, two-tailed, n = 10). Data presented as mean values ± S.E.M .

Supplementary Figure 6

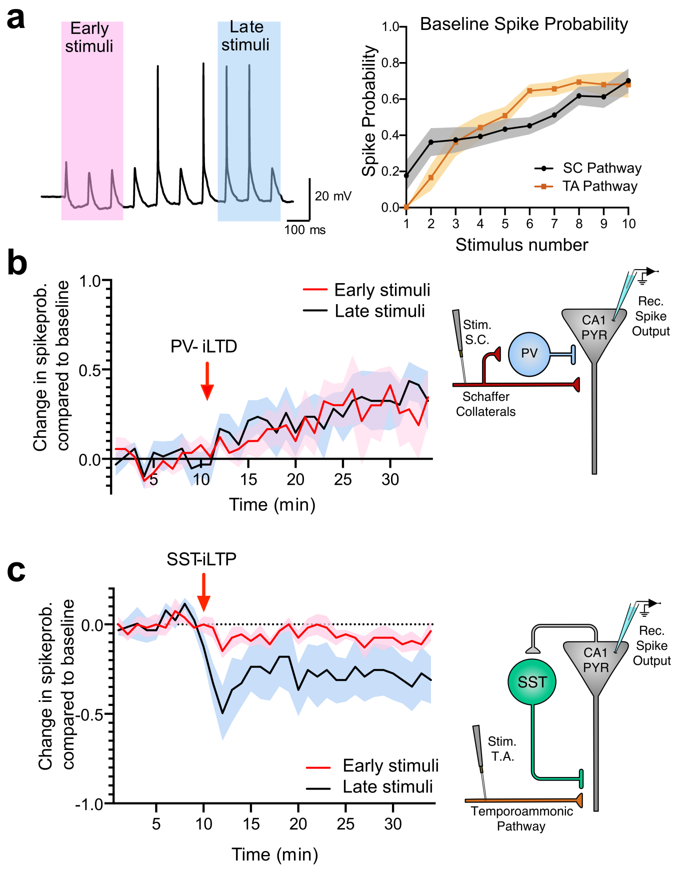

Supplementary Figure 6 (related to Fig. 7). PV and SST plasticity differentially regulate CA1 output to early versus late stimuli within each train.

(a) Example response to a train of stimuli indicating responses classified as early and late. Spike probability for individual stimulated responses - spike probability is lower for earlier responses in both SC and TA pathways, (SC pathway; n = 14 cells, TA pathway; n = 12 cells). **(b)** Probability of spikes in response to early and late stimuli in the SC pathway increases equally after PV-iLTD, (n = 5 cells). **(c)** Probability of spikes in response to late stimuli in the TA pathway decreases more substantially than early spikes in response to SST-iLTP, (n = 6 cells). Data presented as mean values ± S.E.M.

Supplementary Figure 7

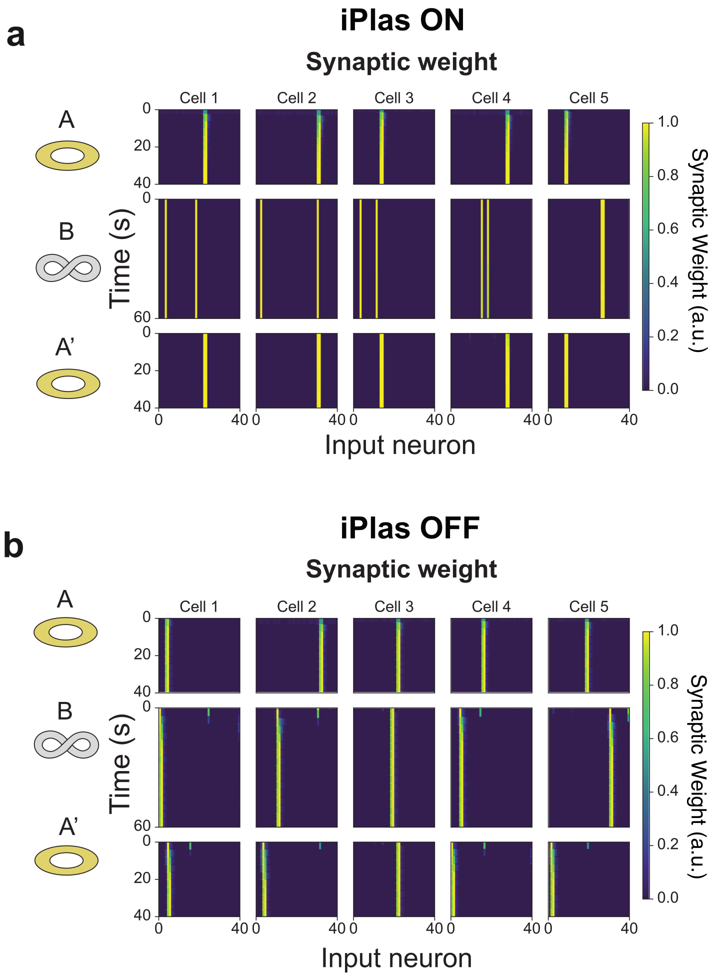

Supplementary Figure 7 (related to Fig. 8). Simulated synaptic weight evolution during exploration in different environments.

(a) Evolution of synaptic weights over time for the example cells shown in Fig. 8b. During these simulations, iPlas is active. **(b)** Evolution of synaptic weights over time for the example cells shown in Fig. 8c. During these simulations, iPlas is turned off.
