# Supplementary material for: Interneuron-specific plasticity at parvalbumin and somatostatin inhibitory synapses onto CA1 pyramidal neurons shapes hippocampal output
Source: Nat Commun. 2020 Sep 2;11:4395. doi: 10.1038/s41467-020-18074-8 (PMC7467931; doi:10.1038/s41467-020-18074-8)
Supplement: Supplementary file 3 — Reporting Summary [file 41467_2020_18074_MOESM3_ESM.pdf]

nature research

Corresponding author(s): Jack R Mellor

Last updated by author(s): Jul 20, 2020

Reporting Summary

Nature Research wishes to improve the reproducibility of the work that we publish. This form provides structure for consistency and transparency in reporting. For further information on Nature Research policies, see Authors & Referees and the Editorial Policy Checklist .

Statistics

For all statistical analyses, confirm that the following items are present in the figure legend, table legend, main text, or Methods section.

n/a	Confirmed	
☐	☒	The exact sample size (*n*) for each experimental group/condition, given as a discrete number and unit of measurement
☐	☒	A statement on whether measurements were taken from distinct samples or whether the same sample was measured repeatedly
☐	☒	The statistical test(s) used AND whether they are one- or two-sided<br>*Only common tests should be described solely by name; describe more complex techniques in the Methods section.*
☒	☐	A description of all covariates tested
☒	☐	A description of any assumptions or corrections, such as tests of normality and adjustment for multiple comparisons
☐	☒	A full description of the statistical parameters including central tendency (e.g. means) or other basic estimates (e.g. regression coefficient) AND variation (e.g. standard deviation) or associated estimates of uncertainty (e.g. confidence intervals)
☐	☒	For null hypothesis testing, the test statistic (e.g. F, t, r) with confidence intervals, effect sizes, degrees of freedom and P value noted<br>*Give P values as exact values whenever suitable.*
☒	☐	For Bayesian analysis, information on the choice of priors and Markov chain Monte Carlo settings
☒	☐	For hierarchical and complex designs, identification of the appropriate level for tests and full reporting of outcomes
☒	☐	Estimates of effect sizes (e.g. Cohen's d, Pearson's r), indicating how they were calculated

Our web collection on statistics for biologists contains articles on many of the points above.

Software and code

Policy information about availability of computer code

Data collection	Electrophysiology data was acquired via Signal v5.12 (CED) software<br>Computational modeling were implemented in python v3.7 using standard libraries
Data analysis	Electrophysiology data was analysed via Signal v5.12 (CED), Graphpad v8 (Prism) and Matlab vR2019a,<br>Image analysis was performed using ImageJ v1.51r<br>Computational modeling was analysed using the code found on ModelDB (accession number 259481).

For manuscripts utilizing custom algorithms or software that are central to the research but not yet described in published literature, software must be made available to editors/reviewers. We strongly encourage code deposition in a community repository (e.g. GitHub). See the Nature Research guidelines for submitting code & software for further information.

Data

Policy information about availability of data

All manuscripts must include a data availability statement. This statement should provide the following information, where applicable:

- Accession codes, unique identifiers, or web links for publicly available datasets
- A list of figures that have associated raw data
- A description of any restrictions on data availability

Further information and data that support the findings of this study are available from the corresponding author Jack.mellor@bristol.ac.uk or upon reasonable request. Computational modelling data is generated by the simulation code (see code availability statement)

Field-specific reporting

Please select the one below that is the best fit for your research. If you are not sure, read the appropriate sections before making your selection.

☒ Life sciences ☐ Behavioural & social sciences ☐ Ecological, evolutionary & environmental sciences

For a reference copy of the document with all sections, see nature.com/documents/nr-reporting-summary-flat.pdf

Life sciences study design

All studies must disclose on these points even when the disclosure is negative.

Sample size	Sample size was calculated as n=6 via power calculations using expected effect size and variability based on previous experiments, with power set at 80% and alpha set at 0.05
Data exclusions	For synaptic plasticity experiments data were excluded if the control pathway deviated >50% or the Series resistance deviated >20% from baseline values. These exclusion criteria were defined prior to data analysis.
Replication	For electrophysiology experiments the experimental unit was defined as cell with only one cell recorded per slice. Up to 3 cells were recorded from each animal with an average of 1.6 cells per animal.
Randomization	Synaptic plasticity experiments were conduced in parallel with control experiments and where possible the induction criteria was randomly assigned. For all other electrophysiology experiments where comparison between groups are made, groups were tested in parallel in an interleaved manner.
Blinding	Blinding was not possible due to requirement of experimenter controlled induction protocols, however experiments were conducted with a within cell control used as an exclusion criteria.

Reporting for specific materials, systems and methods

We require information from authors about some types of materials, experimental systems and methods used in many studies. Here, indicate whether each material, system or method listed is relevant to your study. If you are not sure if a list item applies to your research, read the appropriate section before selecting a response.

Materials & experimental systems

n/a	Involved in the study	
☐	☒	Antibodies
☒	☐	Eukaryotic cell lines
☒	☐	Palaeontology
☐	☒	Animals and other organisms
☒	☐	Human research participants
☒	☐	Clinical data

Methods

n/a	Involved in the study	
☒	☐	ChIP-seq
☒	☐	Flow cytometry
☒	☐	MRI-based neuroimaging

Antibodies

Antibodies used	Anti-PV (raised in mouse) from Sigma, CatN0. P3088<br>Anti-SST (raised in goat) from SantaCruz Biotech. CatNO. SC-7819<br>Anti-GFP (raised in rabbit) from FisherThermo CatNO. A11122<br><br>Secondary<br>Alexa- 594 anti-mouse (raised in goat) FisherThermo CatNO. A11032<br>Alexa - 488 anti-rabbit (raised in goat) FisherThermo CatNO. A11008<br>Alexa- 594 anti-goat (raised in donkey) FisherThermo CatNO. A11058
Validation	Antibodies have been used in multiple peer reviewed articles that can be found alongside validation on the manufacturers website.<br>See:<br>anti-PV: https://www.sigmaaldrich.com/catalog/product/sigma/p3088<br>anti-SST: https://www.scbt.com/p/somatostatin-antibody-d-20<br>anti-GFP: https://www.thermofisher.com/antibody/product/GFP-Antibody-Polyclonal/A-11122

Animals and other organisms

Policy information about studies involving animals; ARRIVE guidelines recommended for reporting animal research

Laboratory animals	several genetically modified mice were used:<br>Ai32 mice (Gt(ROSA)26Sortm32(CAG-COP4*H134R/EYFP)Hze Jax Stock number: 024109)<br>PV-Cre (Pvalbtm1(cre)Arbr/J Jax stock number: 017320)<br>SST-Cre (Ssttm2.1(cre)Zjh/J Jax stock number: 013044)<br>or Chrna2-cre (obtained from the Kullander Laboratory) - Leao, R.N., et al. Nat Neurosci 15, 1524-1530 (2012).<br><br>All mice were on a C57BL/6J background and both male and female mice were used between ages of 4-9 weeks.<br><br>Mice were group housed on a standard 12 hour light/dark cycle (lights on at 7am) with a controlled average ambient temperature of 21°C and 45% humidity
Wild animals	study did not involve wild animals
Field-collected samples	study did not involve samples collected from the field
Ethics oversight	All procedures and techniques were conducted in accordance to the UK animals scientific procedures act, 1986 with approval of the University of Bristol ethics committee.

Note that full information on the approval of the study protocol must also be provided in the manuscript.
